# Hematological parameters in relation to age, sex and biochemical values for mute swans (*Cygnus olor*)

**DOI:** 10.1007/s11259-014-9589-y

**Published:** 2014-01-24

**Authors:** B. Dolka, R. Włodarczyk, A. Żbikowski, I. Dolka, P. Szeleszczuk, W. Kluciński

**Affiliations:** 1 Department of Pathology and Veterinary Diagnostics, Faculty of Veterinary Medicine, Warsaw University of Life Sciences (WULS-SGGW), Nowoursynowska 159c St., 02-776 Warsaw, Poland; 2 Department of Teacher Training and Biodiversity Studies, Faculty of Biology and Environmental Protection, University of Lodz, Banacha 1/3 St, 90-237 Lodz, Poland

**Keywords:** Mute swan, Hematology, Blood chemistry, Body mass, Lead

## Abstract

The knowledge of the correct morphological and biochemical parameters in mute swans is an important indicator of their health status, body condition, adaptation to habitat and useful diagnostic tools in veterinary practice and ecological research. The aim of the study was to obtain hematological parameters in relation to age, sex and serum biochemistry values in wild-living mute swans. We found the significant differences in the erythrocyte count, hematocrit, hemoglobin concentration and erythrocyte sedimentation rate in relation to age of mute swans. There were no differences in hematological values between males and females. The leukogram and H/L ratio did not vary by age and sex in swans. Among of biochemical parameters the slightly increased AST, ALP, CK, K, urea, decreased CHOL and TG values were recorded. As far as we know, this is the first study in which the morphometric parameters of blood cells in mute swans were presented. We found extremely low concentration of lead in blood (at subtreshold level). No blood parasites were found in blood smears. The analysis of body mass and biometric parameters revealed a significant differences dependent on age and sex. No differences in the scaled mass index were found. Our results represent a normal hematologic and blood chemistry values and age-sex related changes, as reference values for the mute swan.

## Introduction

In recent years, the increase of mute swan population (*Cygnus olor*) has been observed in many European countries. It is assumed that this species lost its “wild” instinct to avoid human settlements, more often overwinters in the cities, i.a. due to easier access to the food (Wieloch et al. 2004). The clear “domestication” of mute swans, an increase public interest in environmental protection issues, as well as many cases of highly pathogenic avian influenza (HPAI H5N1) in mute swans in Asia, Europe and Africa (2005–2006) (Ottaviani et al. 2010), makes that these birds more often end up in veterinary clinics, rehabilitation centers and zoos, mostly as victims accidents (injuries, fractures) and poisoning (lead toxicosis) or in emaciated state.

Diagnosis of diseases in mute swans requires knowledge of physiology and pathology, to take effective treatment. In avian veterinary praxis the usefulness of blood test as complementary veterinary tools for the diagnosis is wide known. However, the performance of the blood test, obtaining enough samples, as well as the interpretation of the results still pose many difficulties in wild birds. The results of blood examination must be related to reference ranges, physiological standards for the certain bird species (Ritchie et al. 1994; Olsen et al. 2002; Clark et al. 2009; Milani et al. 2012). The hematological values may be affected by time of day, moulting period, nutrition status, environmental conditions and applied treatment (e.g. steroids) (Shave and Howard 1976; Driver 1981; Whittow 1999; Artacho et al. 2007a; Artacho et al. 2007b; Katavolos et al. 2007; Calabuig et al. 2010; Milani et al. 2012).

The blood examination in mute swans is more often performed, because of importance in clinical diagnosis. Measurement of hematological and biochemical parameters is used for the birds health status assessment, monitoring of the condition, facilitate diagnosis of disease, inflammations, intoxications subclinical processes (Driver 1981; Ritchie et al. 1994; Olsen et al. 2002; Cousquer and Patterson-Kane 2006; Artacho et al. 2007a; Katavolos et al. 2007; Clark et al. 2009; O’Connell et al. 2009).

Mute swans are exposed to numerous pathogens, including blood parasites, such as *Haemoproteus* spp., *Plasmodium* spp., *Leukocytozoon* spp., *Trypanosoma* spp. and the microfilariae. Depending on immunological status, age, parasite loads, the hematozoan infection has impact on hematological parameters resulting in anemia, leukocytosis and eosinophilia (Campbell and Ellis 2007; Clark et al. 2009). The heterophil to lymphocyte ratio (H/L) in the peripheral blood is recognized as a reliable indicator of stress in birds, associated with injury, inflammations, reproductive cycles, seasonal changes, environmental pollution (Gross and Siegel 1983; Maxwell and Robertson 1998; Campbell and Ellis 2007). The literature provides incomplete information about H/L ratio in free-living mute swans.

Mute swans are particular sensitive to lead exposure and may serve as sentinel species for toxicological contaminations in the environment (Degernes 2008). The studies have found many route of exposure to lead in this species (Perrins et al. 2003; Bowen and Petrie 2006; Degernes 2008; O’Connell et al. 2009; Turner and Hambling 2012). Lead toxicosis induced significant changes in the values of commonly measured hematologic parameters in waterfowl (Katavolos et al. 2007). Hematology may show hemolytic anemia (mild to moderate) with Heinz bodies, poikilocytosis, polychromasia. The increased serum uric acid concentrations, LDH and total protein could be observed (O’Halloran et al. 1988; Campbell and Ellis 2007; Katavolos et al. 2007; Degernes 2008; O’Connell et al. 2009).

Despite of the hematological reference ranges for swans have been published (O’Halloran et al. 1988; Jennings 1996; Olsen et al. 2002; Artacho et al. 2007b; Milani et al. 2012), standards for free-living mute swans are still limited in the literature. In this study, we investigated hematology and serum biochemistry values as well as blood lead level in wild-living mute swans. Hematological parameters in relation to age and sex in mute swans and normal morphometrics of blood cells were established.

## Material and methods

### Birds

A total of 95 free-living mute swans (*Cygnus olor*) were captured in wild, regularly within 3 years in early spring (March) and summer (July-September) in central Poland (Mazowieckie Province and Łodzkie Province). Most swans (*n* = 84, 88.4 %) were captured during moulting season when they were not able to fly, with exception of a few juveniles catched in March (*n* = 11, 11.6 %). All swans were captured during the day, at fishponds in pen built of net. Catched birds, with tied legs and wings were transported in kayaks from the pen to the dike where ringing, measuring and blood sampling procedures were performed. Sex was determined by cloacal examination, while the age on the basis of plumage coloration (Conover et al. 2000; Brown and Brown 2002; Calabuig et al. 2011). Two individuals (2.1 %; 2/96) belonged colour variety of the species, called Polish Swan (*Cygnus olor immutabilis*) (Bacon 1980). The birds were divided into 3 age-groups: adult birds (*n* = 49, after 2nd/3rd year of life), immature birds (*n* = 33, in 2nd/3rd year of life) and cygnets (*n* = 13, at the 1st year of life). The gender-groups consist of adult and immature swans: males (*n* = 38), females (*n* = 44) (cygnets were not included).

### Body mass and biometric parameters

Body mass was measured to the nearest 0.1 kg using balance scale. The following biometric measurements were taken: fore-arm length, head length, tarsus length and foot-web width. All measurements were done according to methodology developed for mute swan studies (Mathiasson 2005). We used the tape measure (accuracy: 1 mm) for measuring the lengths of fore-arm, head (to bill tip) and foot-web width. The caliper (accuracy: 0.1 mm) was using for measuring the tarsus length.

### Sample collection

Blood samples were collected from the medial metatarsal vein for hematological tests (tubes with EDTA), for biochemistry and in sodium citrate for ESR (erythrocyte sedimentation rate) (Campbell and Ellis 2007). All swans were marked with numbered ornithological ring and released on the same body of water where they were captured.

### Hematological parameters and serum biochemistry values

The assays were performed using hematological methods recommended to be applied in birds (Ritchie et al. 1994; Campbell and Ellis 2007; Clark et al. 2009). Total number of erythrocytes (RBC) and leukocytes (WBC) were analyzed in Neubauer chamber in Natt-Herrick solution. To obtain packed cell volume or hematocrit (Ht) a capillary tubes were centrifuged at 13.000 rpm/min. for 5 min. in microhematocrit centrifuge MPW – 316 (MPW Med. Instruments, Poland). The hemoglobin concentration (Hb) was estimated using the cyanomethemoglobin method after removal of free red cell nuclei by centrifugation (1,000 g for 10 min.). The mean corpuscular volume (MCV), mean corpuscular hemoglobin (MCH), mean corpuscular hemoglobin concentration (MCHC) were calculated using standard formulas (Campbell and Ellis 2007). To determine the erythrocyte sedimentation rate (in 1 h) we used a blood sedimentation system P-3 (Aquisel, S.L., Spain) according to the standard protocol provided with the system. Blood smears were stained according to the May-Grünwald-Giemsa (MGG) technique for determination of differential leukocyte counts and morphological evaluation of all blood cells. The blood differential test measures the percentage of each type of white blood cell (lymphocytes, heterophils, eosinophils, basophils, monocytes) and the H/L ratio were performed by counting a total of 100 leucocytes in each blood smear (1,000× magnification with oil immersion microscopy). Blood cells were measured using a light microscope Olympus BX41 with CellA software (Olympus). Morphometric parameters evaluated were: mean blood cell diameter (MBCD, μm), mean blood cell area (MBCA, μm^2^), mean blood cell perimeter (MBCP, μm), blood cell roundness (BCR) and erythrocyte nuclear roundness (ENR) calculated as perimeter^2^/4π x area; a perfectly circular structure has a roundness value of 1.0 and values >1 indicate irregular shapes. The stained blood smears were also examined for hemoparasitic infection, initially at low magnification (40×) and then at × 100× using an oil immersion microscopy (Olympus).

Serum for biochemical analyses was obtained by centrifugation (at 3,800 rpm 8 min.) and then processed photometrically on semi-automated chemistry analyzer Pointe 180 (Pointe Scientific, Inc., U.S.) to derermine aspartate aminotransferase (AST), alanine aminotransferase (ALT), alkaline phosphatase (ALP), glucose (GLU), creatinine, urea, uric acid (UA), total protein (TP), albumin (ALB), globulin (GLOB), albumin:globumin ratio (A/G), total bilirubin (BITO), creatine kinase (CK), γ-glutamyltransferase (GGT), amylase (AMY), total cholesterol (CHOL), triglyceride (TG), calcium (Ca), phosphorus (P), magnesium (Mg), potassium (K), sodium (Na). Lead (Pb) concentration in serum samples was determined by graphite furnace atomic absorption spectrophotometry (GFAAS) in Analytical Centre at Warsaw University of Life Sciences (WULS-SGGW), Poland.

### Statistical procedures

The results of body mass, biometric parameters and hematology values were presented in relation to age and sex. For all variables means and parameters of variability, i.e. min., max. and standard error of the mean (SEM) were calculated. Comparison of means was based on analysis of variance and Tukey’s procedure of multiple comparisons at 0.05 probability level. Most of the variables follow assumptions of ANOVA i.e. normal distribution and homogeneity of variances. For the variables which not follow these assumptions log-transformed data were used or non-parametric ANOVA (Kruskal-Wallis test) was used (for eosinophiles, basophiles, monocytes). The analyses were performed using Statistica 10 software (StatSoft). Scaled mass index as a condition index (CI) was calculated according Peig and Green (2009). On the basis of correlation coefficients between morphometric measurements and body mass values the strongest correlation was obtained between the foot-web width and body mass (*r* = 0.903, *P* < 0.001). Since, the width of foot-web was used to calculate scaled mass index. On the basis of the regression analysis we calculated b_SMA_ value 2.54 (b_SMA_ = slope/r: 2.297/0.903), which was used to obtain the scaled mass index (Peig and Green 2009). The P-values *P* < 0.05, *P* < 0.01, *P* < 0.001 were assumed respectively as statistically significant (*), highly significant (**) and the strongest significant (***).

## Results

The results of body mass in particular groups of swans were shown in Table 1. Body mass of all examined mute swans was 9.1 ± 0.2 kg (3.6–13.1 kg). There was strong age-difference (*P* < 0.001) in body mass between adults (10.0 ± 0.2 kg), juveniles (8.8 ± 0.3 kg) and cygnets (6.2 ± 0.7 kg). Males were significantly (*P* < 0.001) heavier than females by 1.7 kg. There were no statistically significant age and sex differences in the mean scaled mass index (Table 2), however only the slightly increased values in adults and females were noted. The results of biometric parameters were shown in Table 1. The measurements of structural parameters revealed significant differences between age-groups and sexes.

**Table 1:** Body mass, biometric parameters (x ± SEM, ranges) of the mute swans in relation to age and sex

Parameter	Age-groups of mute swans			*P*-value	Gender-groups of mute swans		*P*-value	Total
	Adults	Juveniles	Cygnets		Males	Females		
Body mass (kg)	10.0 ± 0.2^a^ (7.5–13.1)	8.8 ± 0.3^b^ (6.0–12.4)	6.2 ± 0.7^c^ (3.6–10.9)	<0.001***	10.5 ± 0.2 (7.2–13.1)	8.8 ± 0.2 (6.0–12.4)	<0.001***	9.1 ± 0.2 (3.6–13.1)
Fore-arm (mm)	286.3 ± 2.4^a^ (224.0–310.0)	286.3 ± 3.0^a^ (260.0–330.0)	108.4 ± 8.6^b^ (90.0–130.0)	<0.001***	294.5 ± 3.0 (224.0–330.0)	279.8 ± 1.8 (260.0–314.0)	0.008**	275.2 ± 5.2 (90.0–330.0)
Head (to bill top) (mm)	177.1 ± 1.0^a^ (163.0–194.0)	176.7 ± 1.6^a^ (158.0–190.0)	136.4 ± 2.8^b^ (131.0–146.0)	<0.001***	182.3 ± 0.9 (172.0–194.0)	172.6 ± 0.9 (158.0–190.0)	<0.001***	177.0 ± 0.9 (158.0–194.0)
Tarsus (mm)	113.0 ± 1.2^a^ (104.4–126.2)	112.3 ± 1.4^a^ (98.4–129.4)	89.3 ± 3.3^b^ (82.0–99.0)	<0.001***	116.1 ± 1.2 (105.5–129.4)	109.7 ± 1.1 (98.4–121.2)	<0.001***	110.4 ± 1.3 (82.0–129.4)
Foot-web (mm)	183.1 ± 1.7^a^ (155.0–205.0)	174.4 ± 2.3^b^ (155.0–202.0)	129.6 ± 2.8^c^ (125.0–140.0)	<0.001***	187.3 ± 1.7 (162.0–205.0)	173.1 ± 1.6 (155.0–190.0)	<0.001***	176.4 ± 1.9 (125.0–205.0)

*P < 0.05; **P < 0.01; ***P < 0.001, *NS* not statistically significant

^a, b, c^means with different letter are significantly different among age-groups of mute swans

**Table 2:** Scaled mass index (condition index - CI) in relation to the age and sex of mute swans

Condition index	Age-groups of mute swans			*P*-value	Gender-groups of mute swans		*P*-value
	Adults	Juveniles	Cygnets		Males	Females	
CI ± SEM (range)	9.2 ± 0.2 (7.5–12.9)	9.0 ± 0.2 (7.4–12.2)	8.9 ± 0.5 (8.1–10.8)	NS (0.604)	9.0 ± 0.2 (7.4–11.4)	9.2 ± 0.2 (7.5–12.9)	NS (0.418)

*P < 0.05; **P < 0.01; ***P < 0.001, *NS* not statistically significant

The results of hematology in relation to age and sex were presented in Tables 3 and 4. We identified the differences in hematological parameters only in relation to age. The results of leukogram and H/L (Table 4) showed no significant differences both in age- and gender-groups of swans. No blood parasites were observed in examined blood smears. The morphometric parameters of blood cells were presented in Table 5. The mean blood cell area (MBCA) was greater for monocyte > basophil > heterophil > eosinophil > erythrocyte > lymphocyte. The erythrocyte nucleus constitutes 14.8 % of whole erythrocyte area. Morphologically only erythrocytes have ovoid shape, other blood cells appeared as round cells. However, monocytes were the most pleomorphic of the leukocytes and their appearance within the same blood smear may be quite varied. The levels for blood biochemical values for mute swans were presented in Table 6.

**Table 3:** Hematological parameters (x ± SEM, ranges) for normal mute swans in relation to age and sex

Parameter	Age-groups of mute swans			*P*-value	Gender-groups of mute swans		*P*-value	Total
	Adults	Juveniles	Cygnets		Males	Females		
RBC (x10^12^/l)	2.07 ± 0.04^a^ (1.72–2.43)	2.04 ± 0.08^a^ (1.44–2.73)	1.58 ± 0.18^b^ (1.01–2.44)	0.003**	2.1 ± 0.05 (1.64–2.7)	2.0 ± 0.08 (1.44–2.73)	NS	1.99 ± 0.05 (1.01–2.73)
Ht (%)	34.5 ± 2.7^ab^ (21.0–44.0)	38.1 ± 1.9^a^ (16.0–45.0)	24.0 ± 4.6^b^ (15.0–30.1)	0.032*	36.3 ± 2.2 (16.0–45.0)	36.9 ± 2.4 (21.0–44.0)	NS	35.3 ± 1.6 (15.0–45.0)
Hb (g/dl)	12.7 ± 1.6^a^ (8.5–24.6)	17.4 ± 1.2^b^ (9.3–23.1)	8.7 ± 1.6^a^ (7.0–10.3)	0.014*	15.2 ± 1.3 (9.3–23.1)	16.1 ± 1.7 (8.5–24.6)	NS	15.1 ± 1.0 (7.04–24.6)
WBC (x10^9^/l)	16.2 ± 2.9 (3.5–29.0)	15.5 ± 1.3 (8.0–29.5)	10.0 ± 2.5 (7.5–12.5)	NS	16.7 ± 1.9 (5.5–29.5)	14.8 ± 2.0 (3.5–29.0)	NS	15.3 ± 1.3 (3.5–29.5)
MCV (μm^3^)	169.8 ± 10.9 (118.0–208.8)	173.9 ± 8.0 (94.1–231.6)	196.2 ± 71.2 (125.0–267.3)	NS	172.8 ± 8.8 (94.1–208.8)	171.9 ± 9.4 (118.0–231.6)	NS	174.2 ± 7.1 (94.1–267.3)
MCH (pg)	62.2 ± 7.2 (39.2–109.8)	80.5 ± 6.1 (43.7–135.9)	80.4 ± 21.7 (58.7–102.0)	NS	71.8 ± 5.7 (43.7–105.6)	75.5 ± 8.3 (39.2–135.9)	NS	74.2 ± 4.7 (39.2–135.9)
MCHC (g/dl)	37.9 ± 4.9 (19.8–68.3)	47.6 ± 4.2 (23.3–91.3)	42.6 ± 4.5 (38.1–47.0)	NS	43.8 ± 5.4 (23.3–91.3)	44.1 ± 4.0 (19.8–68.3)	NS	43.9 ± 3.1 (19.8–91.3)
ESR/60 min. (mm)	8.3 ± 2.6^a^ (1.0–13.0)	2.4 ± 0.2^b^ (1.0–4.0)	–	<0.001***	3.3 ± 1.0 (1.0–8.0)	4.0 ± 1.2 (1.0–13.0)	NS	3.8 ± 0.9 (1.0–13.0)

*P < 0.05; **P < 0.01; ***P < 0.001, *NS* not statistically significant

^a, b, c^means with different letter are significantly different among age-groups of mute swans

**Table 4:** Age- and sex-related leukogram (%) and H/L ratio (x ± SEM, ranges) in mute swans

Parameter	Age-groups of mute swans			*P*-value	Gender-groups of mute swans		*P*-value	Total
	Adults	Juveniles	Cygnets		Males	Females		
Lymphocytes	55.3 ± 1.7 (37.0–69.0)	52.2 ± 2.0 (29.0–68.0)	52.9 ± 3.8 (38.0–62.0)	NS	53.4 ± 1.8 (31.0–69.0)	54.0 ± 2.0 (29.0–68.0)	NS	53.6 ± 1.2 (29.0–69.0)
Heterophils	39.0 ± 1.6 (27.0–53.0)	42.1 ± 2.2 (23.0–69.0)	41.0 ± 3.1 (33.0–55.0)	NS	40.9 ± 1.8 (24.0–62.0)	40.2 ± 2.1 (23.0–69.0)	NS	40.6 ± 1.3 (23.0–69.0)
Eosinophils	1.1 ± 0.3 (0.0–4.0)	1.5 ± 0.3 (0.0–5.0)	1.1 ± 0.4 (0.0–3.0)	NS	1.2 ± 0.3 (0.0–5.0)	1.4 ± 0.3 (0.0–4.0)	NS	1.3 ± 0.2 (0.0–5.0)
Basophils	2.4 ± 0.4 (0.0–9.0)	1.9 ± 0.3 (0.0–6.0)	2.6 ± 0.6 (0.0–4.0)	NS	1.8 ± 0.2 (0.0–3.0)	2.6 ± 0.5 (0.0–9.0)	NS	2.2 ± 0.2 (0.0–9.0)
Monocytes	2.2 ± 0.5 (0.0–8.0)	2.4 ± 0.3 (0.0–6.0)	2.4 ± 1.0 (0.0–7.0)	NS	2.7 ± 0.4 (0.0–7.0)	1.9 ± 0.4 (0.0–8.0)	NS	2.3 ± 0.3 (0.0–8.0)
H/L	0.7 ± 0.1 (0.4–1.4)	0.9 ± 0.1 (0.3–2.4)	0.8 ± 0.1 (0.5–1.5)	NS	0.8 ± 0.1 (0.4–2.0)	0.8 ± 0.1 (0.3–2.4)	NS	0.8 ± 0.1 (0.3–2.4)

*P < 0.05; **P < 0.01; ***P < 0.001, *NS* not statistically significant

**Table 5:** Dimensions of blood cells in mute swans (x ± SEM, ranges)

Blood cell	MBCD (μm)	MBCA (μm^2^)	MBCP (μm)	BCR
Erythrocyte	13.9 ± 0.1 × 7.2 ± 0.1 (12.0–15.7 × 5.2–8.5)	78.8 ± 1.0 (59.3–91.5)	34.0 ± 0.2 (30.4–37.3)	1.2 ± 0.0 (1.1–1.4)
- Erythrocyte nuclei	6.4 ± 0.1 × 2.3 ± 0.0 (5.2–7.5 × 1.8–2.9)	11.7 ± 0.3 (7.5–15.5)	14.6 ± 0.1 (11.7–16.7)	ENR 1.5 ± 0.0 (1.2–1.8)
Lymphocyte	7.2 ± 0.3 (3.8–12.1)	43.6 ± 3.4 (11.6–115.8)	22.6 ± 0.9 (12.1–38.1)	1.0
Heterophil	11.6. ± 0.5 (4.3–18.9)	115.2 ± 9.7 (14.5–280.8)	36.4 ± 1.6 (13.5–59.4)	1.0
Eosinophil	9.8 ± 1.0 (3.0–17.8)	93.0 ± 14.8 (7.1–248.3)	30.9 ± 3.0 (9.5–55.9)	1.0
Basophil	13.4 ± 0.7 (5.8–17.1)	145.5 ± 12.6 (26.3–228.6)	42.0 ± 2.1 (18.2–53.6)	1.0
Monocyte	20.9 ± 0.7 (14.5–24.5)	347.4 ± 22.5 (166.0–471.8)	65.6 ± 2.3 (45.7–77.0)	1.0

*MBCD* mean blood cell diameter; *BCA* mean blood cell area; *MBCP* mean blood cell perimeter; *BCR* blood cell roundness; *ENR* erythrocyte nuclear roundness

**Table 6:** Serum biochemical parameters in mute swans (x ± SEM, ranges)

Parameter	*n*	x ± SEM (range)
AST (U/l)	38	115.7 ± 7.8 (57.5–277.7)
ALT (U/l)	35	52.5 ± 5.2 (8.2–184.4)
ALP U/l	36	142.5 ± 17.7 (13.9–525.7)
GLU (mg/dl)	25	168.1 ± 12.6 (37.4–318.2)
Creatinine (mg/dl)	37	0.8 ± 0.03 (0.3–1.0)
Urea (mg/dl)	49	10.3 ± 1.3 (0.6–45.0)
UA (mg/dl)	50	5.5 ± 0.6 (0.6–14.4)
TB (g/l)	53	45.7 ± 1.1 (30.8–68.1)
ALB (g/l)	50	15.2 ± 0.6 (7.2–25.0)
GLOB (g/l)	51	30.5 ± 1.3 (10.9–51.2)
A/G	50	0.6 ± 0.1 (0.2–2.0)
BITO (mg/dl)	37	0.9 ± 0.2 (0.2–5.9)
CK (U/l)	13	1,239.3 ± 173.3 (194.6–2,745.0)
GGT (U/l)	11	22.0 ± 4.5 (2.2–76.8)
AMY (U/l)	31	1,601.4 ± 95.2 (1,088.0–3,355.0)
CHOL (mg/dl)	47	98.80 ± 4.6 (58.8–205.4)
TG (mg/dl)	40	63.7 ± 7.9 (14.3–238.9)
Ca (mg/dl)	50	10.8 ± 0.3 (6.0–16.8)
P (mg/dl)	20	5.0 ± 0.9 (0.3–15.2)
Mg (mmol/l)	32	2.3 ± 0.1 (1.1–4.4)
K (mmol/l)	17	6.8 ± 0.6 (1.8–12.9)
Na (mmol/l)	12	144.3 ± 7.6 (92.2–226.4)
Pb (μmol/l)	23	0.061 ± 0.006 (0.05–0.15)

## Discussion

The swans are the largest members of the order: *Anseriformes*, family: *Anatidae* and belong to the heaviest flying birds in the world. Mute swans can reach body mass over 12.0 kg (Ritchie et al. 1994). In our study the most of birds were weighted during the moulting season and some of them in March, after cold winter, when access to food was limited. According to literature the body mass of waterfowl during the moulting season declines or remains unchanged (Bacon and Coleman 1986; van Dijk and van Eerden 1991; Hohman et al. 1992). The increase and the peak in the body mass were noted in November – December (Bacon and Coleman 1986). In our study, the results of body mass of examined swans were in normal ranges and appropriate for the season similarly to previous works (Bacon and Coleman 1986; van Dijk and van Eerden 1991; Wieloch et al. 2004). Sex differences in body mass and biometric parameters in our material were consistent with findings obtained by other authors, where males of the mute swan were much heavier and larger than females. We concluded that age and sex had a significant influence on the body mass and size of these birds (Bacon and Coleman 1986; Ritchie et al. 1994; van Dijk and van Eerden 1991; Coleman and Coleman 2002; Wieloch et al. 2004; Calabuig et al. 2011). We showed no differences in the scaled mass index between age classes and sexes that could suggest aligned body condition of examined individuals. In opposite to our results, Coleman and Coleman (2002) indicated sex-difference in body condition where males were in better condition than females among breeders and non-breeders. According to mentioned authors in both sexes, breeding birds were in better condition than non-breeding birds. We suggested that body condition of swans could be more influenced by breeding season.

The obtained mean total RBC counts were approached to the lower reference limits estimated for mute swans and generally for swans (O’Halloran et al. 1988; Jennings 1996; Tully et al. 2000; Cousquer and Patterson-Kane 2006). Based on Williams and Trainer (1971), we indicated that RBC values could be related to seasonal trends. Our results were consistent with data presented by Driver (1981), who demonstrated that some hematological parameters may decrease during and after moulting period compared to values recorded prior to moult. The average Ht values were similar to those reported in mute swans by O’Halloran et al. (1988) and by Cousquer and Patterson-Kane (2006), but lower than Ht in trumpeter swan (*C. buccinator*) and tundra swan (*C. columbianus*) (Ritchie et al. 1994; Olsen et al. 2002; Milani et al. 2012). We found statistically significant differences in the hematological values (RBC, Ht, Hb, ERS) between age classes and no sex differences. According to the literature, before maturity there were nearly no sex differences in total RBC count (Whittow 1999; Campbell and Ellis 2007). Similarly to our results, other authors observed no obvious hematological differences between males and females in geese and ducks (Williams and Trainer 1971; Shave and Howard 1976; Mulley 1979; Milani et al. 2012). However, differences between sexes have been previously reported and can reflect a seasonal variation in hematological parameters and body condition of individuals (Whittow 1999; Campbell and Ellis 2007; Clark et al. 2009; Campbell et al. 2010). The average ESR values were consistent with results recorded from other studies (Sturkie and Textor 1978; Ritchie et al. 1994). We suggested that the age-related differences in ESR values were related to differences in red blood cell parameters and stress levels due to the handling and sampling of birds. The total WBC counts were similar to values reported for mute swans and for other swan species (Jennings 1996; Tully et al. 2000; Olsen et al. 2002; Cousquer and Patterson-Kane 2006; Milani et al. 2012). Our results of leukogram indicated that lymphocytes were the predominant leukocytes in the peripheral blood. This phenomenon was observed in the whole family of *Anseriformes* (swans, ducks, geese) and *Galliformes* (chickens, turkeys) (Ritchie et al. 1994; Campbell et al. 2010; Wakenell 2010). In agreement with other authors we noted no significant differences in leukogram (Williams and Trainer 1971; Mulley 1979; Milani et al. 2012). The H/L ratio have been shown to be a valuable tool to measure stress in birds (Gross and Siegel 1983; Maxwell and Robertson 1998; Campbell and Ellis 2007). In this study, the mean H/L ratio was normal and consistent with other authors, where the normal value in birds was estimated less than 1.0 (Gross and Siegel 1983; Campbell et al. 2010). In other hand, the average H/L values were slightly higher than optimal values for poultry, gulls, American black duck, wood duck, mallards and snow goose (Williams and Trainer 1971; Mulley 1979; Campbell and Ellis 2007; Campbell et al. 2010). In our opinion, this result could be connected with birds’ response to stress caused by handling and collecting procedures.

Based on the absence of hemoparasites in the blood smears we excluded the impact of this factor on hematology results and mute swans health. However, the population of swans should be monitored because of seasonal variations.

The morphology of the cellular elements in the blood of mute swans was similar to those of other birds (Ritchie et al. 1994; Campbell and Ellis 2007; Clark et al. 2009; Campbell et al. 2010; Wakenell 2010). In evaluated blood smears, mature erythrocytes of mute swans had more elongated, elliptical shape with centrally located oval nucleus (with very dense chromatin). The typical heterophils had many brick-red, fusiform granules and usually tri-segmented nucleus. We observed that typical basophils were round cells with nonlobed nucleus and darkly staining basophilic granules in the cytoplasm, which often obscured the nucleus. To our knowledge, no information is available about the morphometric ranges of blood cells of mute swans, which makes it impossible to compare our results with other studies. Generally, dimensions of erythrocytes were within the range specified for the birds (Ritchie et al. 1994; Campbell and Ellis 2007; Clark et al. 2009). The size of mute swans-erythrocyte was larger than erythrocyte of *Galliformes* and mallards (Driver 1981; Ritchie et al. 1994; Wakenell 2010). We indicated, that dimensions of heterophils, basophils, eosinophils and monocytes were larger in comparison with other birds species (Ritchie et al. 1994; Campbell and Ellis 2007; Clark et al. 2009).

In general, the results of serum biochemistry were consistent with the literature regarding swans, mute swans and waterfowl (Mulley 1979; Ritchie et al. 1994; Jennings 1996; Tully et al. 2000; Olsen et al. 2002; Cousquer and Patterson-Kane 2006; Calabuig et al. 2010; Milani et al. 2012). The average values of ALT, glucose, creatinine, uric acid, total protein, albumin, globulin, GGT, calcium were within ranges given for swans and mute swans. The mean activity of AST, ALP and concentration of potassium, urea were slightly increased, but not to abnormally high levels (O’Halloran et al. 1988; Jennings 1996; Tully et al. 2000; Cousquer and Patterson-Kane 2006). We suggested, that the increased AST resulted from overloading the muscle in relation to the capture, handling and immobilization during collecting samples. Moreover, the CPK value was close to the upper reference limits and could indicate an exercise-induced increased AST level (Ritchie et al. 1994; Calabuig et al. 2010; Milani et al. 2012). In our study the A/G ratio was approached to the higher reference limits estimated for mute swans, swans and wild geese (Ritchie et al. 1994; Jennings 1996; Tully et al. 2000). The A/G value was close to values reported in wild tundra swans (Milani et al. 2012). In the present study, mute swans yielded lower TG and CHOL concentrations than reported by other authors (Ritchie et al. 1994; Artacho et al. 2007a; Calabuig et al. 2010; Milani et al. 2012). We supposed that the decline in TG and CHOL can arise due to seasonal changes in metabolism and diet (Ritchie et al. 1994; Artacho et al. 2007a).

According to Perrins et al. (2003) the blood lead level ≥1.21 μmol/l was considered as elevated lead levels in the blood in mute swans. In our study, the mean blood lead level did not exceeded the threshold level for this species. Moreover, RBC, Ht, Hb levels and normal blood smears confirmed health status, no lead toxicosis in examined populations. However, exposure to even low environmental levels of lead may have potentially hazardous effects on health and increased the risk of kidney failure. Similarly to O’Connell et al. (2009) we indicated, that our results may be related with season and with natural habitats of examinated swans.

In our study we demonstrated normal hematologic parameters in relation to age, sex and blood chemistry values for wild-living mute swans. To the best of our knowledge, the results presented here are the first in Poland as well as in Central Europe and one of the few in the world. We believed that our results may have practical significance as reference values for the mute swan and indicators of physiological condition, including health status of this species.
